# Sympatric ecological speciation meets pyrosequencing: sampling the transcriptome of the apple maggot *Rhagoletis pomonella*

**DOI:** 10.1186/1471-2164-10-633

**Published:** 2009-12-27

**Authors:** Dietmar Schwarz, Hugh M Robertson, Jeffrey L Feder, Kranthi Varala, Matthew E Hudson, Gregory J Ragland, Daniel A Hahn, Stewart H Berlocher

**Affiliations:** 1 Department of Entomology, University of Illinois, 320 Morrill Hall, 505 S. Goodwin Ave, Urbana, Illinois, 61801, USA; 2 Department of Biological Sciences, PO Box 369, Galvin Life Science Center, University of Notre Dame, Notre Dame, Indiana, 46556-0369, USA; 3 Department of Crop Sciences, University of Illinois, AW-101 Turner Hall, Urbana, Illinois, 61801, USA; 4 Department of Entomology and Nematology, University of Florida, PO Box 110620, Gainesville, Florida, 32611-0620, USA; 5 Department of Biology, Western Washington University, BI 315 MS9160, Bellingham, Washington, 98225, USA

## Abstract

**Background:**

The full power of modern genetics has been applied to the study of speciation in only a small handful of genetic model species - all of which speciated allopatrically. Here we report the first large expressed sequence tag (EST) study of a candidate for ecological sympatric speciation, the apple maggot *Rhagoletis pomonella*, using massively parallel pyrosequencing on the Roche 454-FLX platform. To maximize transcript diversity we created and sequenced separate libraries from larvae, pupae, adult heads, and headless adult bodies.

**Results:**

We obtained 239,531 sequences which assembled into 24,373 contigs. A total of 6810 unique protein coding genes were identified among the contigs and long singletons, corresponding to 48% of all known *Drosophila melanogaster *protein-coding genes. Their distribution across GO classes suggests that we have obtained a representative sample of the transcriptome. Among these sequences are many candidates for potential *R. pomonella *"speciation genes" (or "barrier genes") such as those controlling chemosensory and life-history timing processes. Furthermore, we identified important marker loci including more than 40,000 single nucleotide polymorphisms (SNPs) and over 100 microsatellites. An initial search for SNPs at which the apple and hawthorn host races differ suggested at least 75 loci warranting further work. We also determined that developmental expression differences remained even after normalization; transcripts expected to show different expression levels between larvae and pupae in *D. melanogaster *also did so in *R. pomonella*. Preliminary comparative analysis of transcript presences and absences revealed evidence of gene loss in *Drosophila *and gain in the higher dipteran clade Schizophora.

**Conclusions:**

These data provide a much needed resource for exploring mechanisms of divergence in this important model for sympatric ecological speciation. Our description of ESTs from a substantial portion of the *R. pomonella *transcriptome will facilitate future functional studies of candidate genes for olfaction and diapause-related life history timing, and will enable large scale expression studies. Similarly, the identification of new SNP and microsatellite markers will facilitate future population and quantitative genetic studies of divergence between the apple and hawthorn-infesting host races.

## Background

How new species arise is a fundamental question in biology. Historically, the formation of new species has been studied in a wide variety of organisms whose natural history provides special insight into the ecological and geographic conditions thought to lead to speciation [1]. But in only a tiny subset of these organisms has it been possible to apply the full power of modern genetics - the ability to identify, sequence, and experimentally manipulate any gene in the genome - to the study of reproductive isolation [2]. Recent advances in sequencing technology, however, have begun to break this constraint. Genomic resources can now be obtained, and are being obtained, for the entire wide range of organisms needed to study the multitudinous modes of speciation [1-3].

Here we have applied transcriptome pyrosequencing to an organism proposed to have undergone sympatric, ecological speciation: the apple maggot fly, *Rhagoletis pomonella *(Walsh). A population of this native North American fruit-feeding insect infesting hawthorn (Rosaceae: *Crataegus *spp.) shifted to attack introduced apple (Rosaceae: *Malus pumila*) in about 1860 [4,5], becoming an economically-important crop pest and, almost immediately after, an evolutionary cause célèbre [6]. The central premise of sympatric speciation in *R. pomonella *is that ecological adaptation to apple has resulted in the formation of a new apple-infesting host race of the fly that is partially reproductively isolated from ancestral hawthorn-infesting populations, a critical first step in the process of ecological sympatric speciation.

The last decade has seen an intensified search for "speciation genes" [7,8] responsible for reproductive barriers between taxa. For the apple and hawthorn host races of *R. pomonella*, two traits have been shown to be of particular importance in host adaptation, and, concomitantly, reproductive isolation. The first trait is host fidelity; because *Rhagoletis *flies mate on or near the fruit of their respective host plants, differences in host choice translate directly into mate choice and prezygotic reproductive isolation [9]. Adult flies use volatile compounds emitted from the surface of ripening fruit as key olfactory cues to find and discriminate among host plants [10]. Flies of the hawthorn and apple host races are attracted to the fruit odors of their respective hosts, and even avoid the odors of the other host fruit [10]. Odor attraction has a genetic basis, but it has not yet been possible to map specific olfactory loci in *Rhagoletis *[11]. But because of the relative phylogenetic proximity of Tephritidae to Drosophilidae, candidate loci for odor recognition can be inferred from the extensive research on chemoreception in *D. melanogaste*r [12,13].

The second trait reproductively isolating the apple and hawthorn host races is life-history timing (and in particular, diapause timing). *Rhagoletis *flies are univoltine and typically have just one generation per year. Flies overwinter as diapausing pupae in the soil and emerge the next summer just prior to the peak of host fruit availability. Because the host plants of *R. pomonella *fruit at different times of year and adult flies live for only about a month, the flies must differentially time their life histories to match maximal host fruit availability for mating and oviposition. The apple race has been selected for both deeper initial diapause depth and earlier post winter eclosion [14-16], in response to the 2-3 week earlier fruiting time of domestic apples compared to hawthorns. Differences in diapause timing between the host races have been associated with a series of chromosome inversions thought to contain linked blocks of genes that affect diapause depth and timing, and thereby allochronic mating isolation between the two host races [17]. Specific loci functionally affecting diapause have not yet been identified and mapped within the inversions for *Rhagoletis*, but genes associated with diapause entry, maintenance, and termination have been described for other insects [18-21], providing useful candidate loci for analysis in *Rhagoletis*.

Although host fruit choice and diapause timing are the key isolating traits, other traits may play a role in reproductive isolation in *Rhagoletis*, and could be analyzed given genomic resources for *Rhagoletis*. Among these are genes for differential larval fitness on the two hosts [22] and for host-independent sexual isolation [23]. In addition, because there is great genetic similarity within the *R. pomonella *species complex [24,25], ESTs generated from *R. pomonella *will be useful for the genetic analysis of the entire sibling species complex, including economically-important crop pests like the blueberry maggot *R. mendax *Curran, and the evolutionarily significant "*Lonicera *fly", a recent population of hybrid *R. mendax *× *R. zephyria *origin [26].

Although the two host races are largely reproductively isolated, low rates of gene flow do occur between the two host races at sites where both occur together [27]. Therefore, the *R. pomonella *host races provide an exceptionally clear example of a "divergence with gene flow" process [28,29]. For some chromosome inversions in *R. pomonella *this process can be studied directly, because the forces producing the balance between host adaptation and the erosion of this adaptation by ongoing gene flow have actually been measured instead of just inferred [9,15,30]. But for the more typical case, in *Rhagoletis *and elsewhere, divergence with gene flow can be studied quantitatively only by measuring variation in divergence among large numbers of loci [31]. While allozyme loci [32-34], and more recently several dozen microsatellite marker loci [35], have provided important insights into population structure and ecological speciation in the *R. pomonella *host races, marker density remains low and developing a series of higher density markers, such as single nucleotide polymorphisms (SNPs) would provide greater resolution for studies of divergence with gene flow. Furthermore high densities of SNPs would provide markers for additional studies like classical genetic mapping, analysis of population structure, and association mapping of loci that are involved in differential host adaptation.

Therefore the primary goals of our study were to: 1) characterize a substantial representation of the *R. pomonella *transcriptome, and 2) to use the assembly of numerous short reads into contigs to identify SNP markers for future studies. In addition to meeting these goals, we have preliminarily identified SNPs that differ between the two host races, we have identified clear differences in the abundance of transcripts between developmental stages, even in normalized cDNA libraries, and we have identified several transcripts that suggest interesting patterns of gene loss or gain among the higher flies.

## Results and Discussion

### Overall sequencing

To maximize transcriptome sampling we made individual libraries for larvae, pupae, adult heads, and headless adult bodies, and sequenced each library separately across several 454 plates (Table 1). The combined analysis of all runs produced 239,531 sequences that assembled into 24,373 contigs (basic sequencing summaries are in Additional file 1). The mean length of contigs was 350 bp, which compares well with the means of 332 and 353 in two other recent 454-based transcriptome studies [36,37]. Contig lengths stretched from 100 bp (the lower bound set by the assembly algorithm) to a maximum of 2823 bp. Contig lengths of 100 - 300 bp were most frequent, but contigs > 300 bp still constituted 32% of all contigs (Additional file 2A). The range of number of reads was broad, stretching from 2 to 1820. The distribution was nevertheless strongly skewed towards contigs with few reads. The mean number of reads per contig was 13.92, but the distribution was highly skewed towards lower read numbers per contig with a long tail of contigs with many reads (SD = 47.97 reads/contig). Only 25% of contigs had 10 reads or more, and 2% of contigs had 100 reads or more with 1820 as the highest number of reads/contig (Additional file 2B). After we filtered all remaining singleton sequences for repeat regions a set of 50,112 singletons were left, 25,090 of which were 100 bp or longer. Files containing our raw sequences and quality scores are available for BLAST search at GenBank SRX001885 and SRX001531 (heads), SRX001121 (larvae), SRX001530 (bodies minus heads), and SRX001529 (pupae).

**Table 1:** Host origin, treatment, and number of individuals used for construction of the four stage/tissue specific libraries.

Stage/Tissue	Samples in each library	Host race	N
Larva	L2+L3, in fruit	Apple	16
	L3, migrant	Apple	6
Pupa	3 days at 25°C	Hawthorn	8
	10 days at 25°C	Hawthorn	8
	20 days at 25°C	Hawthorn	8
	22 days at 25°C	Hawthorn	8
	3 days at 4°C	Hawthorn	6
	1 months at 4°C	Hawthorn	6
	3 months at 4°C	Hawthorn	8
	4 months at 4°C	Hawthorn	8
	3 days at 25°C after diapause	Hawthorn	8
	7 days at 25°C after diapause	Hawthorn	8
	24 days at 25°C after diapause	Hawthorn	8
	40 days at 25°C after diapause	Hawthorn	8
Body (no head)	non-diapaused, 3 days after eclosion	Hawthorn	20
	non-diapaused, 10 days after eclosion	Hawthorn	16
	Wild-caught	Apple	20
Head	non-diapaused, 3 days after eclosion	Hawthorn	98
	non-diapaused, 10 days after eclosion	Hawthorn	91
	wild caught	Apple	51

We annotated our assembled pool of *R. pomonella *sequences (contigs and singletons = 100) to the non-redundant *D. melanogaster *protein coding dataset. Our sequences matched 6810 unique *D. melanogaster *proteins with high confidence (≤ e^-5^). This corresponds to ca. 48% of all known *D. melanogaster *structural, protein-coding genes. Assuming that *Rhagoletis *has a similar gene number as such diverse Diptera as *Anopheles *(ca. 13,000 genes), *Drosophila *(ca. 14,000 genes) and *Aedes *(ca. 17,000 genes), our 6,810 protein-coding genes represent between 40% and 52% of the estimated number of *R. pomonella *protein-coding genes. We note that the exact numbers of genes inferred to be sampled in *R. pomonella *may vary somewhat as we obtain a better understanding of alterative splicing, which will be possible with larger transcriptome data sets. Unannotated contig assemblies are available at GenBank EZ116220 - EZ140593. The data, organized into four files, are also available at http://www.life.illinois.edu/berlocher/454_pyrosequencing_files/. Two Excel files contain annotations of contigs matching protein-coding genes ("Rhagoletis_pom_contig_blast.xls") and singletons ("Rhagoletis_pom_single_blast.xls"). Two text files, "Rhagoletis_pom_all_seq.txt" and "Rhagoletis_pom_contig_seq.txt", contain sequences, and contig and uaccno numbers (read identifications from the 454 machine) to allow for coordination of results.

A notable result of the "short length/high copy" output of pyrosequencing is that some transcripts were sequenced as 2 or 3 noncontiguous fragments that mapped to different parts of the respective homologous *Drosophila *protein. Thirty percent of all *Drosophila *genes matched by our ESTs were matches by both multiple singletons and a contig, demonstrating that a sizable proportion of transcripts were sequenced as multiple, independent pieces. Having several separate fragments distributed at multiple places in any transcript can facilitate efforts to develop full-length sequences for transcripts of interest using traditional PCR-based methods, such as RACE.

Our sequenced fragments matched ca. 50% of all known *D. melanogaster *genes; although our relatively stringent cut-off of e-05 for the protein matches with *D. melanogaster *certainly omitted valid matches to some singletons and/or highly divergent genes. For example, some of the alignments to odorant receptors had e-values several orders of magnitudes larger than e-05, but could still be unequivocally identified as odorant receptors due to the unique sequence structure of these proteins. Directed searches for additional candidate transcripts will undoubtedly reveal many more identifications.

**Figure 1:**
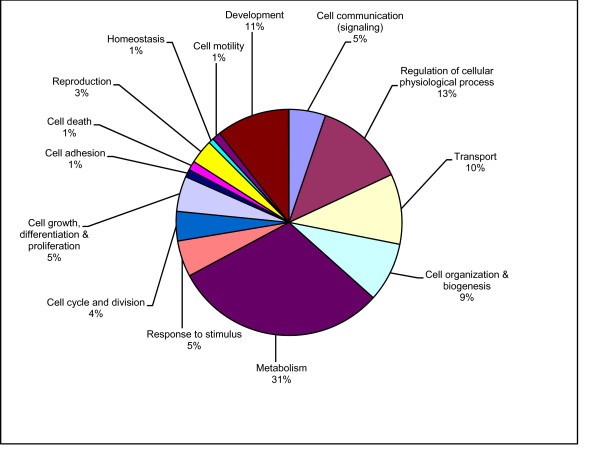
**Annotated *Rhagoletis pomonella *sequences were classified into one of 14 major sub-categories within the Biological Processes GO category**.

**Figure 2:**
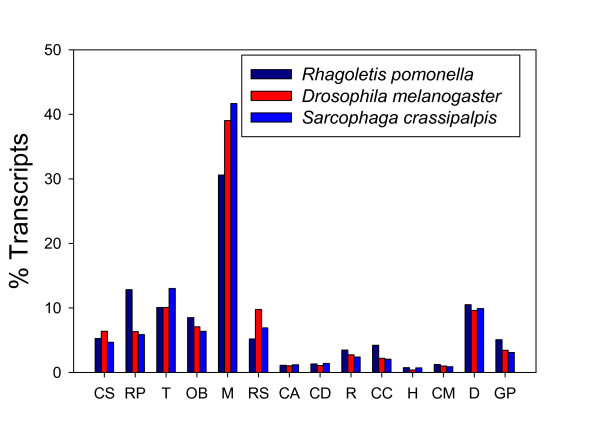
**A comparison of the distribution of ESTs across 14 major Biological Process GO sub-classes in our *Rhagoletis pomonella *library versus predicted ESTs from *Drosophila melanogaster *and ESTs from another higher fly, *Sarcophaga crassipalpis***. The sub-categories are CS = cell communication (signalling), RP = Regulation of cellular physiological process, T = Transport, OB = Cell organization and biogenesis, M = Metabolism, RS = Response to stimulus, CA = Cell adhesion, CD = Cell death, R = Reproduction, CC = Cell cycle and division, H = Homeostasis, CM = Cell motility, D = Development, and GP = Cell growth, differentiation, and proliferation.

A total of 5,666 of our 6,810 annotated transcripts were assigned to 14 major sub-categories in the Biological Process GO category (Fig. 1). To determine if the transcripts we identified were representative of our expectations for the transcriptome as a whole, we compared the distribution of *R. pomonella *sequences mapping to the GO sub-categories described above with similar distributions of transcripts from the entire *Drosophila melanogaster *genome, and the partial transcriptome of the flesh fly *Sarcophaga crassipalpis*, another higher fly for which a substantial EST database has recently been developed using 454-pyrosequencing [36]. While there are small differences in the percentage of transcripts across sub-categories between the three species of flies, none were significantly under- or over-represented across the three species (all Pearson's χ^2 ^p > 0.05). Therefore, the overall concordance between the distributions across all three species suggests that the sequences generated in this project are broadly representative of the *R. pomonella *transcriptome (Fig. 2). Therefore, we have achieved our first major goal of describing a substantial, representative portion of the *R. pomonella *transcriptome.

### Candidate transcripts for host fruit discrimination and diapause, two mechanisms of reproductive isolation between the host races

In addition to our relatively stringent bulk annotations (≤ e^-5^), we specifically searched our contigs and singletons for transcripts that we expected to be involved in host odor discrimination and diapause, two traits contributing to reproductive isolation between the two host races. In searching for receptors, we made use of unique features of receptors (conserved amino acid positions, transmembrane domains, etc.), and in some cases we were able to identify receptors for which the e-value was substantially greater than e^-5^. In our search for chemoreception transcripts, we identified contigs or singletons representing 7 odorant receptors (ORs), 2 gustatory receptors (GRs), and 2 of the newly described [38] ionotropic glutamate receptors (IRs), with multiple fragments for some transcripts (Table 2). All *R. pomonella *putative ORs were represented by a single read with the exception of one matching *D. melanogaster Or 49b*, sequenced in two separate, non-overlapping reads. The *R. pomonella *receptor ESTs were short and ranged in size from 116-283 bp (33-82 aa). If *R. pomonella *has a similar number of ORs to *D. melanogaster*, we estimate that we recovered sequences from about 10% of the ORs [13].

**Table 2:** Contigs and single reads of *Rhagoletis pomonella *candidate chemoreceptors†.

Match	CG	**aa**.	%I	bp	Read/Contig	Source
OR 22c	15377	33	51.0	116	E7OMS0H04JIN70	P
OR 43a	1854	82	53.7	249	E7OMS0H02EYJ4G	H
OR 49a	13158	43	44.0	283	EZ4BI6301E5Z3B*	B
OR 49b	1758	76	65.8	231	C11063 (2)	H
OR 83a	10612	66	51.5	244	E7OMS0H02EEYGV	H
OR 94a	17241	34	52.0	134	E7OMS0H01BOR2 M	B
OR 94b	6679	58	43.1	214	E7OMS0H04H6HYT	P
IR 25a	15627	85	90.0	266	EZ4BI6301FWMUT‡	B
		78	77.0	280	EZ4BI6301EI8 MK	B
IR 92a	15685	38	76.0	247	EZ4BI6301FTL6Z	B
GR 43a	1712	80	60.0	242	E7OMS0H02EBJ2S	H
		37	56.8	146	EY1FUWY01BWJL1	H
		19	85.0	290	EZ4BI6301FFA7L	B
GR 64b	32257	21	66.7	222	E3CVG0K02EHCM3	H

†Contigs and reads matching the same *D. melanogaster *locus map to different regions of the *Drosophila *gene. *Match *is the *D. melanogaster *locus name for the closest match, *CG *is the Celera Genome number of the match, *aa *is the number of amino acids in the single read or contig, *%I *is the percent aa match between the *R. pomonella *and *D. melanogaster *homologous proteins, *bp *is the base pair length of the single read or contig, *Read/Contig *is the *R. pomonella *identifier in our data base, *GenBank *is the GenBank accession number, and *So*. is source (Larva, Pupa, Head, or Body). Number of reads assembled in contig is in parenthesis.

*Also matches an EST fragment of an OR from a congener, *R. suavis *(ABW80750.1) at 100% I; see also GenBank EU204908.1.

‡A second fragment, EZ4BI6301FZM98, was identical but shorter (contained entirely within EZ4BI6301FWMUT).

One of our ORs is homologous to *RSOr1*, previously sequenced in an antennal EST library from the walnut husk fly *R. suavis *using conventional cloning/Sanger sequencing [39]. We did not find matches to the two other tephritid fly ORs reported thus far, both recovered from the Medfly *Ceratitis capitata *using conventional cloning/Sanger sequencing of a head EST library [40]. One of these, the non-canonical *D. melanogaster Or83b*, is highly conserved throughout the insects [41] and its absence in *Rhagoletis *would be surprising. However, one of the major problems in searching for OR sequences is their very low level of expression; in *Drosophila*, previous work found only about one OR per 500,000 clones in an antennal library [42] using conventional cloning/Sanger sequencing. Given this challenge, it is worthwhile to compare approaches for recovering tephritid ORs. The recovery of 1 OR in 544 EST sequences in the *R. suavis *study by Ramsedell et al. [39] suggests that starting with antennae is very effective - but it is also very labor intensive, and was not used in this study for that reason. A previous study describing ORs from Medflies [40] and ours both started with mRNA from adult heads, but the two studies differ in sequencing methodology. The roughly four times higher capture of receptor transcripts in our study (8 vs. 2 for Medfly) is almost certainly due to the due to the higher number of ESTs that are generated by a pyrosequencing approach (239,531 reads for *R. pomonella *vs. 21,253 reads for the Medfly). But pyrosequencing fragments are typically shorter than conventional Sanger sequences, and all of our fragments represent 30% or less of the receptor length as estimated by alignment with *D. melanogaster*. Recovery of sequences of interest was similarly low for IRs and GRs. For example we found only two GRs, which represents only ca. 3% of the 66 GRs that have been discovered in *D. melanogaster*. Ligands have been identified for *Drosophila *homologues of two of the eight odorant receptors identified in this study. Both *Or43a *and *Or49a *respond to compounds typical of fruit odors [13]. Although none of these compounds match fruit volatiles identified thus far as cues for *R. pomonella *[43,44], the *D. melanogaster *work suggests that *Or43a *and *49a *may well be involved in fruit odor detection and therefore are good candidates for future work on host fruit discrimination.

Our recovery rate was much better for the highly expressed OBPs (Table 3). The 22 *R. pomonella *OBPs we discovered in this study correspond to 43% of the 51 known number of *D. melanogaster *OBPs. Alignment with *D. melanogaster *sequences showed that we obtained complete coding sequences for many *R. pomonella *OBP ESTs. We also found cases in which a single *D. melanogaster *OBP represented the best match to two different *R. pomonella *ESTs, as is also the case in *R. suavis *[39], indicating either gene loss in *D. melanogaster *or a duplication in the *Rhagoletis *lineage after the last common ancestor between drosophilids and tephritids.

**Table 3:** Contigs and single reads of *Rhagoletis pomonella *odorant binding proteins (OBPs) and other candidate transcripts for odor reception†.

Match	ID	Aa	%I	bp	Read/Contig
OBP 19a	11748	105	63.8	602 (15)	C10486 [EZ126705]
OBP 19b	2297	120	41.6	578 (29)	C21814 [EZ138033]
OBP 44a	2297	125	65.	934 (159)	C21478 [EZ137697]
OBP 49a	30052	40	50.0	213 (4)	C02098 [EZ118317
OBP 50e	13939	43	41.8	233 (3)	C15401 [EZ131620]
		51	49.0	264	E7OMS0H01BS27Q
OBP 56a	11797	94	27.6	436 (70)	C23516 [EZ139735]
OBP 56d	11218	123	38.2	532 (35)	C00020 [EZ116239]
OBP 56 h	13874	112	37.5	642 (73)	C22766 [EZ138985]
OBP 59a	13517	47	63.8	215	E7OMS0H02EEDPG
OBP 83cd	15582	126	47.6	886 (20)	C20125 [EZ136344]
OBP 83ef	31557	217	49.7	1395 (71)	C20870 [EZ137089]
OBP 83 g	31558	59	57.6	474 (44)	C20023 [EZ136242]
OBP 99b	7592	123	53.6	536 (244)	C19484 [EZ135703]
OBP 99c	7584	139	57.5	759 (76)	C22673 [EZ138892]
OBP 99d	15505	51	45.1	613 (10)	C02834 [EZ119053]
Pbprp 1*	10436	42	50.0	286 (21)	C23956 [EZ140175]
		30	46.0	190 (11)	C22750 [EZ138969]
Pbprp 2	1668	150	25.0	800 (52)	C23271 [EZ139490]
Similar to Pbprp 2*	1668	106	38.6	520 (73)	C14712 [EZ130931]
Pbprp 3*	11421	18	72.0	241 (2)	C02940 [EZ119159]
		144	68.0	410 (4)	C08103 [EZ124322]
Pbprp 4*	1176	124	54.8	737 (60)	C22809 [EZ139028]
Pbprp 5*	6641	128	34.3	820 (136)	C22963 [EZ139182]
Similar to Pbprp 5*	6641	63	44.4	351 (6)	C16946 [EZ133165]
Sensory neuron membrane protein 1	7000	81	75.0	257	E7OMS0H01CAV8S
		92	73.0	276 (5)	C07451 [EZ123670]
G protein salpha 60A	2835	274	93.0	1538 (30)	C08446 [124665]
Arrestin 2	5962	168	97.0	807 (91)	C15900 [EZ132119]
		56	89.0	265	E7OMS0H02EWNVX
Arrestin 1	5711	260	92.0	1306 (79)	C00173 [EZ116392]
Pherokine 3	9358	113	66.0	442 (21)	C02839 [EZ119058]
Putative chemosensory protein CSP1	30172	93	75.0	485 (25)	C23468 [EZ139687]
Cytochrome P450 reductase	11567	140	83.0	1253 (80)	C22056 [EZ138275]

†Contigs and reads matching the same *D. melanogaster *locus map to different regions of the *Drosophila *gene. *Match *is the *D. melanogaster *locus name for the closest match, *CG *is the Celera Genome number of the match, *aa *is the number of amino acids in the single read or contig, *%I *is the percent aa match between the *R. pomonella *and *D. melanogaster *homologous proteins, *bp *is the base pair length of the single read or contig (number of sequences contributing to contig), *Read/Contig *is the *R. pomonella *ID in our data base and the GenBank TSA Accession number.

*Homologous sequence also found in the congener *R. suavis*.

We also discovered fragments of two gustatory receptors (GRs) homologous to *Drosophila Gr43a *and *Gr64b *(Table 2). To our knowledge these are the first GR sequences described for a tephritid fly. Ligands have been described for only a small proportion of the 68 *D. melanogaster *GRs, but among them are the polycistronic *Gr64 *sugar receptors [45] that we also recovered from *R. pomonella *heads. The role of contact chemoreception is largely unstudied in *Rhagoletis *but could be important in detecting differences in fruit surface compounds [46], in the chemical composition of the fruit during oviposition [46], and in host-independent mate choice [23]. All of these factors and processes could act as reproductive barriers between *Rhagoletis *species. Comparative molecular evolutionary analysis of ORs and GRs suggests that these chemoreceptors are correlated with host specialization in *D. sechellia *and *D. erecta *[47], two monophagous specialist species within the mostly polyphagous melanogaster species complex. Characterizing olfactory and gustatory transcripts in *Rhagoletis *provides an opportunity to assess the role of chemoreception in the divergence of a group of phytophagous insects with an extensive radiation into diverse hosts [48].

Although genomic regions correlating with diapause timing have been identified in *R. pomonella *[15,16], specific loci functionally affecting diapause have not yet been identified and mapped in this species. However, genes associated with diapause entry, maintenance, and termination have been described for other insects [18-21], providing useful candidates for analysis in *Rhagoletis*. We generated a list of 92 candidate transcripts by compiling the sets of candidate genes identified by Denlinger et al. [18] and Hahn et al. [36] with transcripts classified as being involved in either "circadian rhythm" or "eclosion rhythm" in the GO matches above. We identified a subset of 45 of these 92 candidate transcripts involved in several major physiological pathways associated with diapause including stress proteins, nutrient storage and metabolism, and endocrine signaling (Additional file 3). Like the chemoreception candidates above, these transcripts provide a substantial resource for functional studies of diapause in *R. pomonella*.

### Marker development

**Figure 3:**
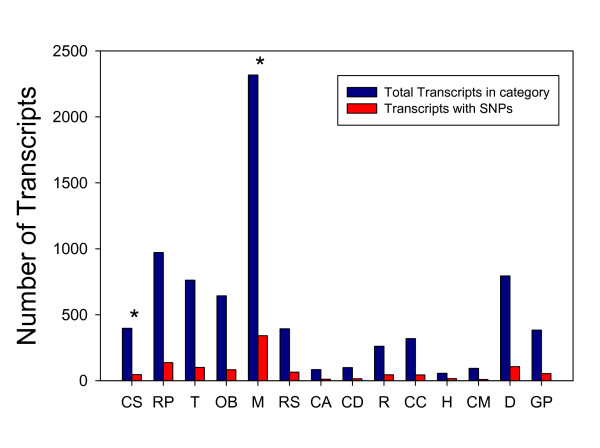
**A comparison of the distribution of SNPs across 14 major Biological Process GO sub-classes in our *Rhagoletis pomonella *library**. The sub-categories are CS = cell communication (signalling), RP = Regulation of cellular physiological process, T = Transport, OB = Cell organization and biogenesis, M = Metabolism, RS = Response to stimulus, CA = Cell adhesion, CD = Cell death, R = Reproduction, CC = Cell cycle and division, H = Homeostasis, CM = Cell motility, D = Development, and GP = Cell growth, differentiation, and proliferation. * = GO categories with slight, but statistically significant, under- or overrepresentation of SNPs (see text).

We also achieved our second major goal, identifying a broad panel of single nucleotide polymorphisms (SNPs) for *R. pomonella*. In total we identified 41,841 SNPs distributed across 5581 contigs. SNPs were approximately randomly distributed across Biological Process GO categories (Fig. 3) with slight underrepresentation within Cell signalling (χ^2 ^= 6.3 p < 0.05) and overrepresentation within Metabolism (χ^2 ^= 10.7 p < 0.001). These SNP locations provide a wealth of information for development of high-throughput downstream population and quantitative genetic applications such as bead chip panels or pull-down marker enrichment strategies followed by direct sequencing. This broad-based approach will allow us to identify regions of genomic similarity and differentiation between interbreeding host races and closely-related species, thereby identifying divergent regions that may house "speciation genes", and testing current models of divergence with gene flow [49].

Because all pupae in this experiment came from a hawthorn population while all larvae came from an apple population, we can also make some initial inferences about SNP genotypes that differ among apple and hawthorn populations. We identified 386 SNPs distributed across 220 contigs that demonstrated allele frequency differences among the host races in our samples. Of the 220 contigs, 126 were annotated (≤ e^-5^), against the *Drosophila melanogaster *genome, although only 77 had characterized assignments within the GO biological process functional category (Additional file 4). We were able to assign reading frames to 76 of the 126 annotated contigs containing 115 total SNPs. Of these SNPs, 64 (56%) were nonsynonymous. However, our confidence in the reading frame is highest within the local BLASTX alignments, and of 42 SNPs located in these regions only 18 (43%) were nonsynomymous (Additional file 5). The latter percentage suggests that there may be quite substantial variation among the host races at the protein coding level. Several of the transcripts containing among host-race SNPs play important roles in metabolism, stress responses, and signalling processes that we expect are important in the diapause response [18,19], including pyruvate kinase, HSP 23 and 27, Superoxide Dismutase II, and Juvenile Hormone Esterase (JHE) binding protein and Malic Enzyme (ME). Of these we were able to identify nonsynonymous SNPs in JHE binding protein and ME. Malic enzyme is one of the original set of allozyme markers used to genetically discriminate the host races [33,34]. One of the two SNPs in ME falls within the local BLASTX peptide alignment with *Drosophila *Malic Enzyme at residue 756, in the first codon position, and produces a change from a polar (Threonine) to a nonpolar (Alanine) amino acid. Six 454 reads from the apple population (all with "A" alleles) and 6 from the haw population (all with "G" alleles) cover the SNP. The Malic Enzyme allozyme polymorphism (for which the allele sequences have not yet been determined) is tightly associated with host-race differences in seasonality [15,16] and may warrant future functional studies given our independent identification of host-specific non-synonymous polymorphism in an unguided analysis. Admittedly, our inferences about host-race specific differences in SNP frequencies in this study are limited because our sampling of the two host races was uneven with the hawthorn population only represented by pupae and the apple population only represented by larvae. However, the host-race specific SNPs identified in this study represent a pool of strong candidates for future validation.

Pyrosequencing EST databases can also identify other sequences useful for population and quantitative genetic studies, such as microsatellite loci [36]. Using MSATCOMMANDER [50] we conservatively identified 169 microsatellite repeats among our contigs and singletons (Additional file 6). We have not yet formally assessed any of these loci for variability, but they may be a useful supplement to a panel of 80 GT-enriched microsatellites that have previously been developed [51] and successfully used for studies of *R. pomonella *population structure [35].

### Expression differences in larval and pupal transcripts

Despite normalization, we obtained some information about stage-specific expression from our separately sequenced stage libraries (larva, pupa, adult heads, and adult bodies minus heads). We point out at the onset that we are not suggesting that accurate measurement of expression differences can or should be performed on normalized libraries, only that large, essentially "on or off" expression information can be obtained in the very first transciptome study on a species by tagging or separately sequencing different stages or tissues. To illustrate the potential for finding such large stage-biased expression differences, we compared a subset of transcripts in our larval and pupal pools that we expected would have larval-biased expression. These included larval cuticle transcripts [52] and transcripts for the digestive enzyme trypsin; neither should be produced during the non-feeding pupal stage [53]. Our panel of larval cuticle genes (12 contigs annotated to 8 different *D. melanogaster *cuticle transcripts) showed the expected bias with 893 out of 938 reads (95%) occurring in larval samples (Table 4). Similarly, of the 7 trypsin-related contigs, 411 out of 446 reads (92%) occurred in larval samples. The percentage of these transcripts expressed in the larva would undoubtedly be greater in non-normalized RNA, but the unequal expression pattern we found is quite clear.

**Table 4:** Sequences expressed primarily in larvae†.

Contig	Total reads	Larval reads	% L reads	Match	Annotation
*Larval Cuticle Proteins*					
17158 [EZ133377], 24176 [EZ140395], 9309 [125528]	175	173	99	CG32400	Larval cuticular protein 65Ab1
17157 [EZ133376]	38	37	97	CG6956	Larval cuticular protein 65Ac
9308 [EZ125527], 9306 [EZ125525]	177	171	97	CG2044	Larval cuticular protein 4
22477 [EZ138696]	29	28	97	CG15515	Cuticle protein
11180 [EZ127399]	82	79	96	CG9070	Larval cuticular protein 2a
21675 [EZ137894], 24313 [EZ140532]	308	290	94	CG8697	Larval cuticle protein 2
17322 [EZ133541]	34	32	94	CG9077	Cuticular protein 47Ec
20182 [EZ136401]	95	83	87	CG8502	Cuticular protein 49Ac
*Protein Digestion*					
23604 [EZ139823]	36	36	100	CG12385	theta-Trypsin
24013 [EZ140232]	48	46	96	CG12385	theta-Trypsin
10762 [EZ126981]	89	85	96	CG12385	theta-Trypsin
21083 [EZ137302]	54	49	91	CG17571	trypsin-like serum protease
9347 [EZ125566]	120	108	90	CG30028	gammaTrypsin
9544 [EZ125763]	79	70	89	CG12385	theta Trypsin
22598 [EZ138817]	20	17	85	CG12387	zeta Trypsin

†"Contig" is our assembly number followed by the GenBank TSA accession number, "total reads" is the total number of reads contributing to the contig, "larval reads" is the number of the total recovered from larvae, "%L" is the percent of reads from larvae, "Match" is the Celera Genome number of the best match with *Drosophila melanogaster*, and "Annotation" is a brief description of the match.

### Estimates of gene loss and gain in comparison with *Drosophila*

We identified two transcripts, for the genes Armadillo repeat-containing-protein 8 and Cellular retinaldehyde-binding protein, for which the evidence strongly supports gene loss in *Drosophila*. Protein BLAST search revealed that *R. pomonella *contig 11078 (EZ127297, 502 amino acids, about 73% complete inferred from comparison with the 681 aa in the homologous complete *Culex quinquifasciatus *sequence) was very similar to the Armadillo repeat-containing-protein 8 locus of highly divergent organisms ranging from the mosquito *Culex quinquifasciatus *(XP_001861563.1, I = 51%, 1e-143), *Homo sapiens *(EAW79075.1, I = 39%, 3e-104), the placozoan *Trichoplax adhaerens *(XP_002110308.1, I = 34%, 2e-52), and the fungus *Ajellomyces dermatitidis *(EEQ71730.1, I = 29%, 9e-13). Yet the best match for *R. pomonella *contig 11078 to a gene in the *D. melanogaster *complete genome was to Beta Adaptin (CG12532, NP_523415.1, I = 23%), at an e value of 0.52. Armadillo repeat-containing-protein 8 is a single copy gene in all sequenced insect genomes except for *Aedes aegyptii *(2 copies). Similar results were obtained for contig 23434 (EZ139653) which matched the Cellular retinaldehyde-binding protein locus throughout the Animalia, but did not produce a high match within *D. melanogaster*.

Gene gains were also implied; we identified eight transcripts that had not been previously identified as unique to the clade Schizophora (containing *Drosophila*, *Rhagoletis*, and house flies and their ilk). For four of these (contig 24366 [EZ140585], 01397 [EZ117616], 10054 [EZ126273], and 09054 [EZ125273], all of unknown function) we found no matching non-Schizophoran sequences at all. For the remaining four novel genes (contigs 09325 [EZ125544], 04014 [EZ120233], 01753 [EZ117972], and 04040 [EZ120259]), we were able to identify distant ancestors outside the Schizophora; some clearly represent new members of multigene families (Additional file 7).

Our inferences about gene gain and loss are admittedly limited because an EST project produces an incomplete sample of a species' genes. For example, we cannot determine whether the absence of a gene in *Rhagoletis *is due to the actual absence of this gene, or to sampling error. However, we can conclude with confidence that a gene has been lost in *Drosophila *when it occurs in *Rhagoletis *and other insects but is missing in the completely sequenced genomes of *Drosophila melanogaster *and relatives. Similarly, we can only be certain about whether an apparently *Drosophila*-specific gene was gained prior to the common ancestor of *Rhagoletis *and *Drosophila*, but not about whether it was gained more recently after the drosophilid/tephritid split. Even with these sampling limitations, however, gene gain/loss information from transcriptome studies has great potential for resolving the genealogies among *Rhagoletis*, *Drosophila*, and other Schizophora.

## Conclusions

The transcriptome data reported here will greatly expand our insight into all the areas to which *Rhagoletis pomonella *has contributed: ecological speciation, sympatric speciation, host plant adaptation, and the emergence of new economically important insect pests. The impact of the new data will first be felt in studies of olfaction and diapause-related life history timing, for which this study has provided a wealth of new candidate loci. But the SNP and microsatellite markers reported here will also facilitate new work on the population and quantitative genetic studies of divergence between the apple and hawthorn-infesting host races. Furthermore, these data will serve as a basis for exploring the molecular genetics of host plant radiation and adaptation more broadly in the closely-related members of the *R. pomonella *species group, which contains a bevy of host-plant specialists including the economically-important blueberry maggot *R. mendax *and the newly identified "*Lonicera *fly," an example of hybrid speciation.

## Methods

All stages except egg and first instar larvae were included in our libraries. We collected 2^nd ^and 3^rd ^instar larvae infested apples from a fallow orchard in Urbana, IL during summer 2007 by dissecting them from apples, washing in water, and freezing at -80°C. We reared pupae from infested hawthorn fruit collected from trees in South Bend, IN, in fall 2006. Some pupae were transferred to 4°C after a prediapause period of two weeks and kept under diapause conditions for 4 months, while a second set of pupae was kept at 25°C until eclosion. Pupae were frozen from each set at regular intervals. After removal from diapause additional pupae from the first set were reared at 25°C to provide hawthorn race adults. This stratified sampling plan allowed us to collect a variety of developmental stages including prewinter diapausing pupae, postwinter diapausing pupae, and multiple points of pharate adult development [54] and post eclosion adult maturation. Apple host race adults were caught on fruit in a fallow apple orchard in Urbana, IL, in the summer of 2007. Adult heads were separated from the rest of the body, with the goal of increasing representation of olfactory transcripts, and heads and bodies were used to construct separate libraries. For numbers of flies representing each stage/body part/host race in our libraries and how they were spread across sequencing runs see Table 1.

We used a two-step RNA extraction procedure beginning with an initial TRIZOL (Invitrogen) extraction, followed by further purification on the filter-based RNeasy (Qiagen) kit. Beginning the extraction with TRIZOL extraction maximizes clean yields from fatty tissues, particularly in larvae and pupae, and the filter-based RNeasy kit eliminated the genomic DNA and body pigments typically left behind by TRIZOL extraction. Total RNA extractions were pooled into four samples representing 1) larvae, 2) pupae, 3) adult bodies minus heads, and 4) adult heads. The Oligotex Mini Kit (Qiagen) was then used to purify mRNA from each of the four total RNA pools. cDNAs were synthesized from 500 ng of mRNA following the Clontech's Creator SMART cDNA synthesis system using modified Oligo-dT and 5' RACE primers. Primer sequences were: CDSIII-First 454: 5' TAG AGA CCG AGG CGG CCG ACA TGT TTT GTT TTT TTT TCT TTT TTT TTT VN 3' and SMARTIV: 5' AAG CAG TGG TAT CAA CGC AGA GTG GCC ATT ACG GCC GGG 3'.

Separate normalization of each of the four stage-specific cDNA libraries and 454 pyrosequencing were carried out at the W.M. Keck Center for Comparative and Functional Genomics, University of Illinois at Urbana-Champaign, Roy J. Carver Biotechnology Center. For normalization, 300 ng of cDNAs were denatured, allowed to self-anneal, DSN treated (Duplex/double stranded specific Nuclease; Evrogen, Russia), and remaining transcripts were PCR amplified to make normalized ds-cDNAs. Titration runs were performed for each of the four libraries on 1/16^th ^region of a 70 × 75 PicoTiterPlate (PTP), and subsequent bulk runs were split between two 1/4^th ^regions respectively to spread each bulk run across at least two plates (Additional file 1).

Sequence assembly was performed using the Newbler Assembler with sequences masked for oligonucleotide adaptors used in SMART cDNA synthesis and normalization. All contigs and singlets were annotated by BLAST search against both the *Drosophila melanogaster *non-redundant protein database where the e-value threshold was set at 1e^-5 ^for confident annotation. Gene Ontology (GO) assignments were also assigned based on sequence similarity to *D. melanogaster *and transcripts with GO assignments were collapsed down into 14 major GO categories under the Biological Process category.

To specifically search for transcripts associated with olfaction, and therefore possibly host fruit choice, we searched our contigs and long (>100 bp) singleton sequences with complete sets of *D. melanogaster *odorant receptors (ORs), gustatory receptors (GRs), and odorant binding proteins (OBPs), and recorded all hits to all of these receptors, even in cases of relatively large e-values. We then evaluated all hits manually for the presence of characteristic conserved amino acids, transmembrane domains, and other features of olfactory and gustatory receptors. We similarly searched for several other transcripts from *Drosphila *or moth species that have been implicated in peripheral chemoreception (K. Wanner, personal communication). The regulation of diapause is much less well understood, but we compiled and searched our data for a set of candidate transcripts identified by Denlinger et al. [18] and Hahn et al. [36]. We further searched for transcripts classified as being involved in either "circadian rhythm" or "eclosion rhythm" in the GO matches above, yielding a set of 92 total candidate transcripts for diapause. EST sequences matching a gene from these transcripts of interest were aligned (BLASTX) against the NCBI NR protein data set to verify best match to our EST. This methodology does not ensure that the gene of interest and the *Rhagoletis *EST are orthologs or even fulfil the same function, but does provide a first hypothesis for future functional studies.

Because multiple individuals from different populations were combined for the pyrosequencing run we can identify putative single nucleotide polymorphism (SNP) variation within and between sequencing runs. To accomplish this we re-assembled reads in MOSAIK http://bioinformatics.bc.edu/marthlab/Mosaik using the Newbler-assembled contigs as anchors. Newbler assembly notation codes putative SNPs with flanking insertion characters (presumably to reflect uncertainty in indel errors), whereas most reference-guided assemblies provide a direct alignment at polymorphic positions that are easily recognized by SNP-finding packages. We used GigaBayes http://bioinformatics.bc.edu/marthlab/GigaBayes[55,56] to estimate the (Bayesian) posterior probability that a given variable site in the re-assembly represents a true polymorphism, setting the threshold probability at 90%.

Because pupae and larvae were run separately and represent pools from hawthorn and apple populations, respectively, we were also able to identify putative host-specific allelic variation in SNPs. We calculated Pearson χ^2 ^statistics for a host race by allele contingency table at each SNP locus, and we report host specific alleles as those with χ^2^_1 _values exceeding p = 0.01. This test is not strictly applied because multiple reads could have come from the same individual, resulting in a non-representative population sample. However, the procedure provides a useful metric to identify candidates for future confirmation/characterization. In addition, we established reading frames from local BLASTX alignments [37] using the methods of Hahn et al. [36] to compensate for 454 under/overcall errors. From these alignments we determined whether each SNP putatively segregating between host races is synonymous or nonsynonymous. We performed conservative formal searches for microsatellite motifs within our sequences using the program MSATCOMMANDER [50] to identify sequences containing di-, tri-, tetra-, penta-, and hexanucleotide repeats with a minimum length of 7.

To determine whether stage specific expression can still be detected in normalized libraries, we focused on subsets of contigs annotated as larval cuticle or digestive transcripts, both of which are, in other species, expressed only in larvae [52,53]. We considered only contigs with ≥ 20 reads in order to examine whether we would be able to detect strongly skewed contributions of larvae or pupae to a given contig.

Although any EST project is unlikely to represent all of a species' transcriptome, EST data can provide important first pass on transcript gain or loss among taxa. We used information from previous analyses that identified groups of orthologous genes (ortholog groups) by comparing completed genomes using Smith-Waterman sequence comparisons. The results from these analyses are available in an online data base (orthoDB) that allows searching for ortholog groups according to level of conservation in different taxonomic groups [57]. To test for ortholog groups that were lost in *Drosophila *but potentially retained in *Rhagoletis*, we compiled a list of loci that had a single copy in *Apis*, *Tribolium*, *Bombyx*, *Anopheles*, *Aedes*, and *Culex*, but were not found in any of the 12 annotated *Drosophila *genomes (n = 66 ortholog groups). To identify genes that are unique to both *Rhagoletis *and *Drosophila *we limited our search to genes that had at least one copy in each of the 12 *Drosophila *species but were absent in non-drosophilid genomes (n = 850 ortholog groups). All nucleotide sequences from these ortholog groups were blasted against our set of *Rhagoletis *contigs using the tBLASTn algorithm. Translated amino acid sequences of contigs that had a blast match with an expected value < = e-30 to one or more translated sequences from a particular ortholog groups were aligned with all the translated amino acid sequences from that group with ClustalW. These alignments were imported into the MEGA software, which allowed for Genbank searches of the contig using the BLASTp algorithm (local alignment of protein sequences) against the non-redundant protein database (NCBI-NR). We displayed all results of these BLAST searches using the web-BLAST's built-in construction of neighbour-joining phylogenetic trees. If this first screening supported the respective hypothesis of gene loss or gain, then the top BLAST hits down to suitable outgroups were imported into MEGA and aligned with the *Rhagoletis *contig and ortholog group sequences using ClustalW. We trimmed the alignment to match the aligned length of the *Rhagoletis *sequences and constructed phylogenetic trees (neighbour-joining, 1000 bootstrap intervals). In order to infer the presence of a gene in *Rhagoletis *that is absent in *Drosophila *we required that the translated *Rhagoletis *sequence formed a well-supported clade with outgroup species genes (bootstrap values > 70) that did not include any *Drosophila *copies. To infer that a gene was novel to both *Rhagoletis *and *Drosophila*, it had to 1) form a well supported clade (bootstrap value > 70) with its closest *Drosophila *relative that lacked any non-drosophilid genes and 2) form a separate sister clade to a related sister clade composed of *Rhagoletis *and *Drosophila *genes. Our detection scheme was conservative because it ignored cases in which there was no evidence for an ancestral gene in the Schizophora [58,59]. However, it avoided the quandary of having to decide at which point along a single evolutionary branch a sequence represented a new gene. The only exception for this rule was made in cases when no insect matches other than *Drosophila *could be obtained from Genbank. In this case the gene in question was classified as novel to the Schizophora.

## Competing interests

The authors declare that they have no competing interests.

## Authors' contributions

DS, SHB, HMR, and JLF conceived the study and design and collected samples. DS and HMR made the libraries and prepared samples for sequencing. DS, SHB, HMR, MWH, GJR, KV, and DAH participated in the data analysis. DS, SHB, HMR, MWH, GJR, DAH and JLF drafted the manuscript. All authors read and approved the final manuscript.

## Supplementary Material

Additional file 1 **Supplemental table**. Table of sequencing scheme and summary statistics for titration and bulk runs. Click here for file

Additional file 2 **Descriptive figures of contig lengths and coverage**. 2a. Distribution of contig lengths. 2b. Coverage (number of reads per contig) by contig length. Click here for file

Additional file 3 **Table of candidate ESTs for diapause regulation and emergence timing**. Contigs and reads matching the same *D. melanogaster *locus map to different regions of the *D. melanogaster *gene. *Match *is the *D. melanogaster *locus name for the closest match, *CG *is the Celera Genome number of the match, aa is the number of amino acids in the single read or contig, *%I *is the percent aa match between the *R. pomonella *and *D. melanogaster *homologous proteins, *bp *is the base pair length of the single read or contig (number of sequences contributing to contig), *Read/Contig *is the *R. pomonella *ID in our data base. Click here for file

Additional file 4 **Table of contigs containing SNPs that differed in frequency between the two host races**. Contig is the R. pomonella contig number followed by the TSA accession number. CG is the D. melanogaster Celera Genome number of the locus with the closest match and Annotation is the D. melanogaster locus name where known. Click here for file

Additional file 5 **Table of listing synonomous/nonsynonomous changes in contigs containing SNPs that differed in frequency between the two host races**. For those contigs where an open reading frame could be clearly identified, we determined whether SNPs would affect the amino acid sequence of the protein product. Contig is the R. pomonella contig number followed by the TSA accession number. CG is the D. melanogaster Celera Genome number of the locus with the closest match and Annotation is the D. melanogaster locus name where known. Position denotes the nucleotide location within the contig and synonymous? denotes whether the alternative forms of the SNP specify alternative amino acids. We also define the consensus amino acid, the alternative amino acid, the consensus codon and whether the SNP site is within the local BLAST alignment of our data with the hit to the identified D. melanogaster locus. Click here for file

Additional file 6 **Table describing microsatellite discovery**. 6a. Summary of potential microsatellite loci identified. 6b. List of contigs and singletons containing potential microsatellite loci including repeat type and length. Click here for file

Additional file 7 **Table of transcript gain in the lineage leading to the Schizophora since the last common ancestor of mosquitoes, Rhagoletis, and Drosophila**. D. melanogaster annotation denotes the Celera Genome number of the locus with the closest match and locus name where known. Contig is the R. pomonella contig number followed by the TSA accession number. Click here for file
