# Supplementary material for: Sympatric ecological speciation meets pyrosequencing: sampling the transcriptome of the apple maggot Rhagoletis pomonella
Source: BMC Genomics. 2009 Dec 27;10:633. doi: 10.1186/1471-2164-10-633 (PMC2807884; doi:10.1186/1471-2164-10-633)
Supplement: Additional file 1 — Supplemental table. Table of sequencing scheme and summary statistics for titration and bulk runs. [file 1471-2164-10-633-S1.DOC]

**Additional file 1**

| **Sample** | **Plate Type** | **Num. Reads** | **Total Bases** | **Avg. read length** |
| --- | --- | --- | --- | --- |
|  |  |  |  |  |
| **Bulk runs** |  |  |  |  |
| Body | 1/1 25x75_LR | 55,348 | 13,721,932 | 247.92 |
|  | 1/4 70x75_LR | 10,928 | 2,777,766 | 254.21 |
|  | 1/4 70x75_LR | 54,976 | 14,205,000 | 258.39 |
| **Body total** |  | **121,252** | **30,704,698** | **253.23** |
|  |  |  |  |  |
| Head | 1/4 70x75_LR | 68,522 | 16,712,928 | 243.91 |
|  | 1/4 70x75_LR | 35,908 | 8,324,085 | 231.82 |
|  | 1/4 70x75_LR | 44,717 | 10,235,000 | 228.90 |
| **Head total** |  | **149,147** | **35,272,013** | **236.49** |
|  |  |  |  |  |
| Larva | 1/4 70x75_LR | 20,843 | 4,874,366 | 233.86 |
|  | 1/4 70x75_LR | 44,827 | 10,487,000 | 233.94 |
| **Larva total** |  | **65,670** | **15,361,366** | **233.92** |
|  |  |  |  |  |
| Pupa | 1/4 70x75_LR | 24,867 | 5,323,860 | 214.09 |
|  | 1/4 70x75_LR | 30,721 | 6,354,000 | 206.83 |
| **Pupa total** |  | **55,588** | **11,677,860** | **210.08** |
|  |  |  |  |  |
| **Bulk runs total** |  | **391,657** | **93,015,937** | **237.49** |
|  |  |  |  |  |
|  |  |  |  |  |
| **Titration runs** |  |  |  |  |
| Head | ~1/4 70x75_SR | 7,240 | 792,551 | 109.05 |
| Larva | ~1/4 70x75_SR | 8,974 | 989,960 | 111.17 |
| Pupa | ~1/4 70x75_SR | 6,901 | 745,905 | 107.90 |
| **Titration runs total** |  | **23,115** | **2,528,416** | **109.38** |
