# Supplementary figures and images for: Sympatric ecological speciation meets pyrosequencing: sampling the transcriptome of the apple maggot Rhagoletis pomonella
Source: BMC Genomics. 2009 Dec 27;10:633. doi: 10.1186/1471-2164-10-633 (PMC2807884; doi:10.1186/1471-2164-10-633)

Additional file 2A


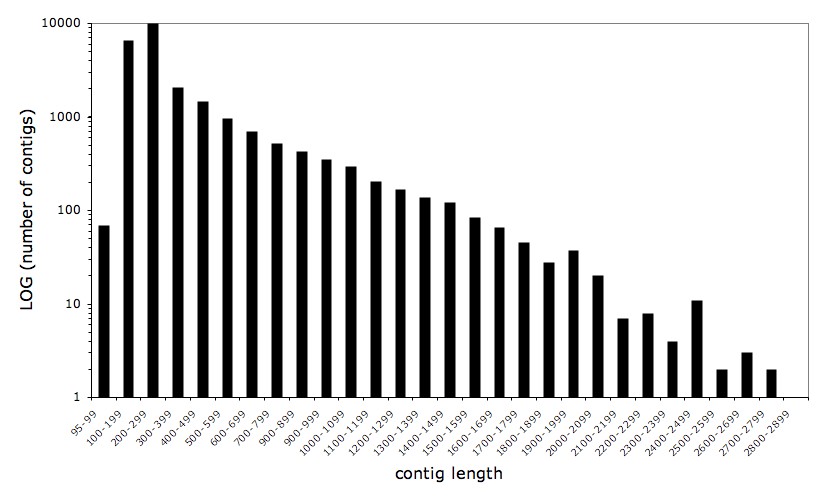


Additional file 2B


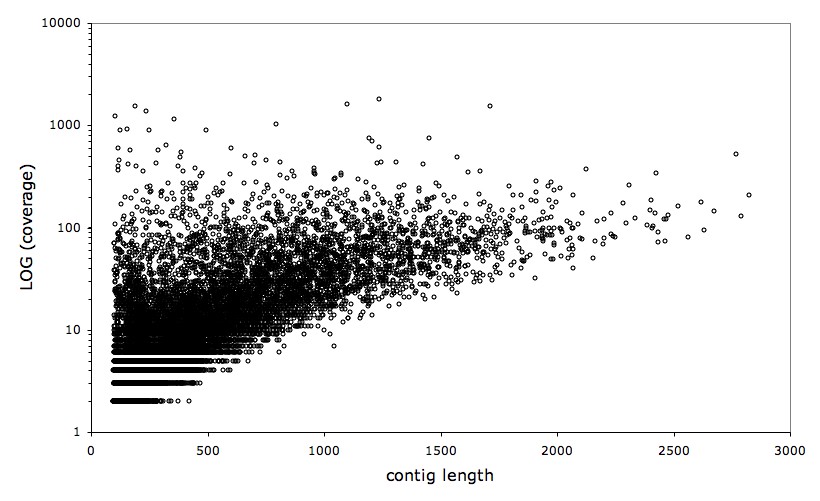

Supplement: Additional file 2 — Descriptive figures of contig lengths and coverage. 2a. Distribution of contig lengths. 2b. Coverage (number of reads per contig) by contig length. [file 1471-2164-10-633-S2.DOC]
