# Supplementary material for: Sympatric ecological speciation meets pyrosequencing: sampling the transcriptome of the apple maggot Rhagoletis pomonella
Source: BMC Genomics. 2009 Dec 27;10:633. doi: 10.1186/1471-2164-10-633 (PMC2807884; doi:10.1186/1471-2164-10-633)
Supplement: Additional file 3 — Table of candidate ESTs for diapause regulation and emergence timing. Contigs and reads matching the same D. melanogaster locus map to different regions of the D. melanogaster gene. Match is the D. melanogaster locus name for the closest match, CG is the Celera Genome number of the match, aa is the number of amino acids in the single read or contig, %I is the percent aa match between the R. pomonella and D. melanogaster homologous proteins, bp is the base pair length of the single read or contig (number of sequences contributing to contig), Read/Contig is the R. pomonella ID in our data base. [file 1471-2164-10-633-S3.DOCX]

**Additional file 3**

| **Match** | **CG** | **aa** | **%I** | **bp** | **Read/Contig** | **TSA acc.** |
| --- | --- | --- | --- | --- | --- | --- |
| Ras oncogene at 85D | 9375 | 130 | 94 | 852 (23) | C03807 | EZ120026 |
| *Insulin like reception* |  |  |  |  |  |  |
| gigas | 6975 | 80 | 90 | 241 (2) | C17873 | EZ134092 |
|  |  | 66 | 76 | 444 (7) | C09477 | EZ125696 |
| Dock | 3727 | 112 | 90 | 547 (8) | C10646 | EZ126865 |
|  |  | 25 | 100 | 222 (3) | C04606 | EZ120825 |
|  |  | 78 | 65 | 232 (2) | C11966 | EZ128185 |
| melted | 8624 | 43 | 95 | 210 (2) | C07309 | EZ123528 |
| Chico | 5686 | 88 | 41 | 269 | E7OMS0H01BDIG9 |  |
|  |  | 48 | 39 | 236 (4) | C22515 | EZ138734 |
| Tsc1 | 6147 | 79 | 75 | 241 (3) | C09819 | EZ126038 |
|  |  | 72 | 71 | 241 (2) | C13526 | EZ129745 |
| Foxo | 3143 | 56 | 62 | 197 | EZ4BI6301FQGAJ |  |
| Ilp4 | 6736 | 51 | 33 | 251 | E3CVG0K02C8VJM |  |
|  |  | 54 | 35 | 317 (4) | C14779 | EZ130998 |
| Insulin precursor¥ | 14173 | 104 | 28 | 417 (5) | C01648 | EZ117867 |
| Pi3K92E | 4141 | 169 | 61 | 487 (6) | C10126 | EZ126345 |
|  |  | 79 | 87 | 241 (3) | C13241 | EZ129460 |
|  |  | 80 | 55 | 261 | E3CVG0K01B9R49 |  |
|  |  | 32 | 97 | 110 | E7OMS0H01BIZPR |  |
| Ras oncogene at 85D | 9375 | 130 | 94 | 852 (23) | C03807 | EZ120026 |
|  |  |  |  |  |  |  |
| *Protein Storage* |  |  |  |  |  |  |
| Larval serum protein 1 gamma | 6821 | 59 | 84 | 197 (2) | C04414 | EZ120633 |
|  |  | 79 | 58 | 243 | E7OMS0H03F0MKH |  |
|  |  | 74 | 45 | 224 | E7OMS0H03FSY4J |  |
| Larval serum protein 2 | 6806 | 378 | 63 | 1254 (174) | C22607 | EZ138826 |
| regucalcin | 1803 | 299 | 74 | 956 (82) | C23657 | EZ139876 |
| garnet | 10986 | 86 | 76 | 262 | E7OMS0H03G9XDG |  |
| Putative hexamerin |  |  |  | 230 | E7OMS0H04I3PPC |  |
|  |  |  |  |  |  |  |
| *Juvenile hormone* |  |  |  |  |  |  |
| Jhe | 8425 | 483 | 41 | 1983 (49) | C20630 | EZ136849 |
| Jheh1 | 15101 | 258 | 58 | 893 (40) | C20499 | EZ136718 |
|  |  | 182 | 61 | 988 (28) | C21749 | EZ137968 |
| Jheh2 | 15102 | 77 | 74 | 241 (3) | C12959 | EZ129178 |
|  |  | 66 | 74 | 271 | E3CVG0K04I48VS |  |
| Jheh3 | 15106 | 81 | 71 | 247 | E7OMS0H03FL18J |  |
|  |  | 85 | 58 | 263 | E7OMS0H03GIOPT |  |
|  |  |  |  |  |  |  |
|  |  |  |  |  |  |  |
| *Metabolic depression* |  |  |  |  |  |  |
| Glycogen phosphorylase | 7254 | 327 | 93 | 1435 (64) | C10098 | EZ126317 |
|  |  | 73 | 95 | 222 | E7OMS0H01A9GL8 |  |
|  |  | 70 | 90 | 216 | E7OMS0H03G7S6V |  |
|  |  | 76 | 84 | 230 | E7OMS0H01A0ATG |  |
|  |  |  |  |  |  |  |
| cAMP-dependent protein kinase 1 | 4379 | 270 | 98 | 1586 (123) | C12635 | EZ128854 |
| cAMP-dependent protein kinase 2 | 12066 | 80 | 62 | 241 | E7OMS0H01CA39F |  |
| cAMP-dependent protein kinase 3 | 6117 | 92 | 89 | 400 (11) | C14089 | EZ130308 |
|  |  | 71 | 90 | 216 (2) | C01429 | EZ117648 |
| *Stress proteins* |  |  |  |  |  |  |
| Cu-Zn SOD† | 9027 | 176 | 64 | 772 (12) | C02136 | EZ118355 |
| HSP 23 | 4463 | 175 | 60 | 780 (116) | C18808 | EZ135027 |
|  |  | 120 | 73 | 956 (52) | C20615 | EZ136834 |
|  |  | 116 | 73 | 361 (69) | C23482 | EZ139701 |
| HSP 27 | 4466 | 220 | 61 | 1011 (72) | C10844 | EZ127063 |
| HSP 68 | 5436 | 135 | 89 | 652 (13) | C00036 | EZ116255 |
|  |  | 137 | 65 | 448 (24) | C21700 | EZ137919 |
| HSP 70Aa | 31366 | 156 | 72 | 749 (27) | C21126 | EZ137345 |
| HSP 70Ba | 31449 | 390 | 66 | 1166 (29) | C21633 | EZ137852 |
| HSP 83 | 1242 | 362 | 92 | 1471 (92) | C11080 | EZ127299 |
|  |  | 206 | 95 | 1247 (32) | C22942 | EZ139161 |
| Glycoprotein 93 | 5520 | 57 | 89 | 173 (2) | C01030 | EZ117249 |
|  |  | 141 | 75 | 447 (10) | C11741 | EZ127960 |
|  |  | 108 | 91 | 805 (45) | C11758 | EZ127977 |
|  |  | 78 | 88 | 235 (3) | C14422 | EZ130641 |
| Hsc 3 | 4147 | 139 | 97 | 690 (17) | C01840 | EZ118059 |
|  |  | 456 | 96 | 1964 (100) | C09596 | EZ125815 |
| Hsc 4 | 4264 | 139 | 95 | 418 (7) | C12213 | EZ128432 |
|  |  | 67 | 95 | 203 (3) | C14646 | EZ130865 |
| Trap1 | 3152 | 271 | 76 | 820 (10) | C19816 | EZ136035 |
|  |  |  |  |  |  |  |
| *Other diapause* |  |  |  |  |  |  |
| Pyrokinin-1 receptor* | 9918 | 54 | 51 | 200 (4) | C14446 | EZ130665 |
| ultraspiracle | 4380 | 135 | 61 | 599 (11) | C09816 | EZ126035 |
|  |  | 325 | 64 | 1188 (46) | C23405 | EZ139624 |
| Similar to sarcocystatin | 15369 | 119 | 49 | 466 (321) | C09028 | EZ125247 |
| G protein salpha 60A | 2835 | 274 | 93 | 1538 (30) | C08446 | EZ124665 |
|  |  |  |  |  |  |  |
| *Circadian/Eclosion* |  |  |  |  |  |  |
| CkIIbeta | 15224 | 189 | 98 | 1469 (102) | C09035 | EZ125254 |
| dopa decarboxylase | 10697 | 67 | 98 | 204 (2) | C18385 | EZ134604 |
|  |  | 81 | 80 | 251 | E7OMS0H01BUQPF |  |
|  |  | 81 | 74 | 579 (19) | C08619 | EZ124838 |
|  |  | 32 | 84 | 260 | EZ4BI6301EDDBK |  |
|  |  | 73 | 72 | 241 (2) | C05157 | EZ121376 |
| lark | 8597 | 80 | 75 | 241 (2) | C06508 | EZ122727 |
| cycle | 8727 | 73 | 82 | 221 | E7OMS0H04H3GU1 |  |
|  |  | 67 | 76 | 248 | E7OMS0H01BZNQD |  |
|  |  | 43 | 93 | 226 (4) | C02481 | EZ118700 |
| timeless | 3234 | 78 | 65 | 431 (5) | C03101 | EZ119320 |
|  |  | 73 | 87 | 235 | E7OMS0H02DDJMG |  |
|  |  | 69 | 47 | 207 | EZ4BI6301EBC0X |  |
|  |  | 21 | 71 | 427 (14) | C08493 | EZ124712 |

*Homologous to diapause receptor 1 in *Bombyx*.

†Homologous to *Bombyx* time-interval measuring enzyme.

¥Closest match to Aedes aegypti AaeL_AAEL000973.
