# Supplementary material for: Sympatric ecological speciation meets pyrosequencing: sampling the transcriptome of the apple maggot Rhagoletis pomonella
Source: BMC Genomics. 2009 Dec 27;10:633. doi: 10.1186/1471-2164-10-633 (PMC2807884; doi:10.1186/1471-2164-10-633)
Supplement: Additional file 4 — Table of contigs containing SNPs that differed in frequency between the two host races. Contig is the R. pomonella contig number followed by the TSA accession number. CG is the D. melanogaster Celera Genome number of the locus with the closest match and Annotation is the D. melanogaster locus name where known. [file 1471-2164-10-633-S4.DOCX]

**Additional file 4**

| **Contig** | **TSA Acc.** | **CG** | **Annotation** |
| --- | --- | --- | --- |
| contig13175 | EZ129394 | CG10120 | Malic enzyme |
| contig09056 | EZ125275 | CG10272 | grappa |
| contig21309 | EZ137528 | CG10621 | CG10621 |
| contig09464 | EZ125683 | CG1065 | Succinyl coenzyme A synthetase alpha subunit |
| contig23923 | EZ140142 | CG10652 | Ribosomal protein L30 |
| contig17173 | EZ133392 | CG10794 | Diptericin B |
| contig18219 | EZ134438 | CG10922 | La autoantigen-like |
| contig22174 | EZ138393 | CG11134 | CG11134 |
| contig20383 | EZ136602 | CG11590 | CG11590 |
| contig17665 | EZ133884 | CG11852 | CG11852 |
| contig20581 | EZ136800 | CG11989 | Ard1 |
| contig18033 | EZ134252 | CG12055 | Glyceraldehyde 3 phosphate dehydrogenase 1 |
| contig22581 | EZ138800 | CG12379 | CG12379 |
| contig24027 | EZ140246 | CG1249 | snRNP2 |
| contig01499 | EZ117718 | CG12756 | Eaf6 |
| contig00725 | EZ116944 | CG12775 | Ribosomal protein L21 |
| contig20572 | EZ136791 | CG13315 | CG13315 |
| contig09219 | EZ125438 | CG13389 | Ribosomal protein S13 |
| contig23936 | EZ140155 | CG13628 | Rpb10 |
| contig00687 | EZ116906 | CG14120 | CG14120 |
| contig22236 | EZ138455 | CG14482 | CG14482 |
| contig23106 | EZ139325 | CG14812 | CG14812 |
| contig10340 | EZ126559 | CG14881 | CG14881 |
| contig24222 | EZ140441 | CG14996 | Chd64 |
| contig20314 | EZ136533 | CG15098 | CG15098 |
| contig22093 | EZ138312 | CG1532 | CG1532 |
| contig08637 | EZ124856 | CG15693 | Ribosomal protein S20 |
| contig23306 | EZ139525 | CG15881 | CG15881 |
| contig02915 | EZ119134 | CG1662 | CG1662 |
| contig09398 | EZ125617 | CG1665 | CG1665 |
| contig09482 | EZ125701 | CG1667 | CG1667 |
| contig21132 | EZ137351 | CG16865 | CG16865 |
| contig21173 | EZ137392 | CG17233 | CG17233 |
| contig21402 | EZ137621 | CG17285 | Fat body protein 1 |
| contig11261 | EZ127480 | CG1742 | Microsomal glutathione S-transferase-like |
| contig20075 | EZ136294 | CG1789 | CG1789 |
| contig22065 | EZ138284 | CG17903 | Cytochrome c proximal |
| contig09943 | EZ126162 | CG17931 | CG17931 |
| contig20669 | EZ136888 | CG18473 | CG18473 |
| contig08926 | EZ125145 | CG1850 | CG1850 |
| contig14676 | EZ130895 | CG1963 | pterin-4a-carbinolamine dehydratase |
| contig09252 | EZ125471 | CG2746 | Ribosomal protein L19 |
| contig23185 | EZ139404 | CG2803 | Troponin C-akin-1 |
| contig23762 | EZ139981 | CG2922 | extra bases |
| contig22540 | EZ138759 | CG2998 | Ribosomal protein S28b |
| contig22926 | EZ139145 | CG30349 | CG30349 |
| contig00888 | EZ117107 | CG30423 | CG30423 |
| contig24247 | EZ140466 | CG31605 | Basigin |
| contig09822 | EZ126041 | CG31717 | CG31717 |
| contig23686 | EZ139905 | CG31751 | CG31751 |
| contig22245 | EZ138464 | CG31778 | CG31778 |
| contig23412 | EZ139631 | CG3186 | eIF-5A |
| contig22405 | EZ138624 | CG3226 | CG3226 |
| contig22406 | EZ138625 | CG3226 | CG3226 |
| contig09309 | EZ125528 | CG32400 | Lcp65Ab1 |
| contig08822 | EZ125041 | CG32744 | Ubiquitin-5E |
| contig22930 | EZ139149 | CG33198 | presenilin enhancer |
| contig09205 | EZ125424 | CG33199 | CG33199 |
| contig13222 | EZ129441 | CG33493 | CG33493 |
| contig23561 | EZ139780 | CG33672 | CG33672 |
| contig22673 | EZ138892 | CG33978 | CG33978 |
| contig24297 | EZ140516 | CG34074 | mitochondrial Cytochrome c oxidase subunit III |
| contig23165 | EZ139384 | CG34117 | CG34117 |
| contig14709 | EZ130928 | CG34183 | - |
| contig22037 | EZ138256 | CG34310 | - |
| contig19484 | EZ135703 | CG34346 | maternal gene required for meiosis |
| contig22086 | EZ138305 | CG3625 | CG3625 |
| contig21642 | EZ137861 | CG3661 | Ribosomal protein L23 |
| contig21160 | EZ137379 | CG3776 | Juvenile hormone esterase binding protein 29 |
| contig23038 | EZ139257 | CG3798 | N-methyl-D-aspartate receptor-associated protein |
| contig20482 | EZ136701 | CG3997 | Ribosomal protein L39 |
| contig20440 | EZ136659 | CG40127 | CG40127 |
| contig23330 | EZ139549 | CG4027 | Actin 5C |
| contig22080 | EZ138299 | CG4111 | Ribosomal protein L35 |
| contig22154 | EZ138373 | CG42325 | Phosphodiesterase 1c |
| contig11178 | EZ127397 | CG4299 | Set |
| contig23586 | EZ139805 | CG4463 | Heat shock protein 23 |
| contig10844 | EZ127063 | CG4466 | Heat shock protein 27 |
| contig20450 | EZ136669 | CG4511 | CG4511 |
| contig21091 | EZ137310 | CG4576 | CG4576 |
| contig00032 | EZ116251 | CG4593 | CG4593 |
| contig20678 | EZ136897 | CG4618 | CG4618 |
| contig23348 | EZ139567 | CG4800 | Translationally controlled tumor protein |
| contig22908 | EZ139127 | CG4882 | CG4882 |
| contig24289 | EZ140508 | CG5271 | Ribosomal protein S27A |
| contig20571 | EZ136790 | CG5378 | Rpn7 |
| contig13734 | EZ129953 | CG5384 | CG5384 |
| contig21109 | EZ137328 | CG5767 | CG5767 |
| contig01239 | EZ117458 | CG6090 | Ribosomal protein L34a |
| contig12506 | EZ128725 | CG6105 | lethal (2) 06225 |
| contig17590 | EZ133809 | CG6188 | CG6188 |
| contig22522 | EZ138741 | CG6207 | GlcAT-P |
| contig22868 | EZ139087 | CG6395 | Cysteine string protein |
| contig17934 | EZ134153 | CG6461 | CG6461 |
| contig23061 | EZ139280 | CG6463 | CG6463 |
| contig21031 | EZ137250 | CG6662 | CG6662 |
| contig23937 | EZ140156 | CG6783 | CG6783 |
| contig23717 | EZ139936 | CG6988 | Protein disulfide isomerase |
| contig20152 | EZ136371 | CG7070 | Pyruvate kinase |
| contig21486 | EZ137705 | CG7181 | CG7181 |
| contig23210 | EZ139429 | CG7283 | Ribosomal protein L10Ab |
| contig12661 | EZ128880 | CG7490 | Ribosomal protein LP0 |
| contig17870 | EZ134089 | CG7626 | Spt5 |
| contig20490 | EZ136709 | CG7641 | Neurocalcin |
| contig00965 | EZ117184 | CG7970 | CG7970 |
| contig21425 | EZ137644 | CG7993 | CG7993 |
| contig12261 | EZ128480 | CG8048 | Vacuolar H[+] ATPase 44kD C subunit |
| contig14735 | EZ130954 | CG8211 | CG8211 |
| contig22629 | EZ138848 | CG8331 | CG8331 |
| contig12368 | EZ128587 | CG8415 | Ribosomal protein S23 |
| contig23633 | EZ139852 | CG8446 | CG8446 |
| contig20182 | EZ136401 | CG8502 | Cuticular protein 49Ac |
| contig20848 | EZ137067 | CG8549 | CG8549 |
| contig11852 | EZ128071 | CG8573 | Suppressor of Hairy wing |
| contig22533 | EZ138752 | CG8759 | Nascent polypeptide associated complex protein |
| contig23946 | EZ140165 | CG8764 | Oxen |
| contig22619 | EZ138838 | CG8905 | Superoxide dismutase 2 (Mn) |
| contig19625 | EZ135844 | CG9240 | CG9240 |
| contig20121 | EZ136340 | CG9401 | mago nashi |
| contig22108 | EZ138327 | CG9494 | Tetraspanin 29Fa |
| contig13831 | EZ130050 | CG9662 | CG9662 |
| contig21078 | EZ137297 | CG9762 | lethal (3) neo18 |
| contig00244 | EZ116463 | - | - |
| contig00250 | EZ116469 | - | - |
| contig20277 | EZ136496 | - | - |
|  |  |  |  |
