# Supplementary material for: Sympatric ecological speciation meets pyrosequencing: sampling the transcriptome of the apple maggot Rhagoletis pomonella
Source: BMC Genomics. 2009 Dec 27;10:633. doi: 10.1186/1471-2164-10-633 (PMC2807884; doi:10.1186/1471-2164-10-633)
Supplement: Additional file 6 — Table describing microsatellite discovery. 6a. Summary of potential microsatellite loci identified. 6b. List of contigs and singletons containing potential microsatellite loci including repeat type and length. [file 1471-2164-10-633-S6.DOCX]

**Additional file 6**

| **6a. Repeat type** | **# of hits (repeat length >7)** | | |
| --- | --- | --- | --- |
|  |  | |  |
| Dinucleotide | 125 |  |  |
| Trinucleotide | 35 |  |  |
| Tetranucleotide | 7 |  |  |
| Hexanucleotide | 3 |  |  |

| **6b. SequenceID** | **TSA Acc.** | **Motif Type** | **Repeat** |
| --- | --- | --- | --- |
| contig08878 | EZ125097 | Dinucleotide | (GT)^7 |
| contig04529 | EZ120748 | Dinucleotide | (AT)^8 |
| contig03705 | EZ119924 | Dinucleotide | (AT)^7 |
| contig15103 | EZ131322 | Dinucleotide | (GT)^7 |
| contig20565 | EZ136784 | Dinucleotide | (AT)^7 |
| contig01242 | EZ117461 | Dinucleotide | (AC)^8 |
| contig02360 | EZ118579 | Dinucleotide | (AT)^8 |
| contig19789 | EZ136008 | Dinucleotide | (GT)^10 |
| contig22876 | EZ139095 | Dinucleotide | (CT)^9 |
| contig20857 | EZ137076 | Dinucleotide | (GT)^13 |
| contig15082 | EZ131301 | Dinucleotide | (AT)^7 |
| contig20753 | EZ136972 | Dinucleotide | (GT)^19 |
| contig19659 | EZ135878 | Dinucleotide | (AT)^7 |
| contig01366 | EZ117585 | Dinucleotide | (AT)^12 |
| contig11374 | EZ127593 | Dinucleotide | (AC)^8 |
| contig15980 | EZ132199 | Dinucleotide | (CT)^7 |
| contig09473 | EZ125692 | Dinucleotide | (AC)^8 |
| contig00630 | EZ116849 | Dinucleotide | (GT)^8 |
| contig01763 | EZ117982 | Dinucleotide | (AT)^9 |
| contig02573 | EZ118792 | Dinucleotide | (GT)^7 |
| contig04653 | EZ120872 | Dinucleotide | (AT)^7 |
| contig01184 | EZ117403 | Dinucleotide | (GT)^7 |
| contig09431 | EZ125650 | Dinucleotide | (AC)^8 |
| contig21355 | EZ137574 | Dinucleotide | (AC)^8 |
| contig20528 | EZ136747 | Dinucleotide | (GT)^8 |
| contig02803 | EZ119022 | Dinucleotide | (AC)^7 |
| E0V4D1301AQP90 |  | Dinucleotide | (AT)^8 |
| contig09741 | EZ125960 | Dinucleotide | (GT)^7 |
| contig14598 | EZ130817 | Dinucleotide | (AC)^7 |
| contig20714 | EZ136933 | Dinucleotide | (AG)^9 |
| contig00713 | EZ116932 | Dinucleotide | (AT)^7 |
| contig23374 | EZ139593 | Dinucleotide | (GT)^8 |
| contig24317 | EZ140536 | Dinucleotide | (AC)^8 |
| contig20446 | EZ136665 | Dinucleotide | (AC)^10 |
| contig05547 | EZ121766 | Dinucleotide | (AT)^7 |
| contig13992 | EZ130211 | Dinucleotide | (AC)^9 |
| contig11846 | EZ128065 | Dinucleotide | (AC)^10 |
| contig01833 | EZ118052 | Dinucleotide | (AT)^7 |
| contig15634 | EZ131853 | Dinucleotide | (AT)^7 |
| contig04380 | EZ120599 | Dinucleotide | (AC)^10 |
| contig01843 | EZ118062 | Dinucleotide | (GT)^7 |
| contig23699 | EZ139918 | Dinucleotide | (GT)^7 |
| contig22193 | EZ138412 | Dinucleotide | (GT)^7 |
| contig21359 | EZ137578 | Dinucleotide | (AC)^7 |
| contig05650 | EZ121869 | Dinucleotide | (AC)^14 |
| contig03555 | EZ119774 | Dinucleotide | (AC)^7 |
| contig03074 | EZ119293 | Dinucleotide | (AT)^8 |
| contig12522 | EZ128741 | Dinucleotide | (AT)^8 |
| contig19337 | EZ135556 | Dinucleotide | (GT)^7 |
| contig04176 | EZ120395 | Dinucleotide | (AT)^7 |
| contig21414 | EZ137633 | Dinucleotide | (AT)^8 |
| contig24374 | EZ140593 | Dinucleotide | (AC)^8 |
| contig19272 | EZ135491 | Dinucleotide | (AC)^8 |
| contig12919 | EZ129138 | Dinucleotide | (GT)^10 |
| contig17460 | EZ133679 | Dinucleotide | (GT)^7 |
| contig22193 | EZ138412 | Dinucleotide | (GT)^11 |
| contig04105 | EZ120324 | Dinucleotide | (AC)^7 |
| contig04534 | EZ120753 | Dinucleotide | (GT)^8 |
| contig05789 | EZ122008 | Dinucleotide | (AG)^8 |
| contig20513 | EZ136732 | Dinucleotide | (AT)^7 |
| contig09036 | EZ125255 | Dinucleotide | (AT)^8 |
| contig08295 | EZ124514 | Dinucleotide | (AT)^9 |
| contig21870 | EZ138089 | Dinucleotide | (AC)^7 |
| contig18285 | EZ134504 | Dinucleotide | (AT)^8 |
| contig22504 | EZ138723 | Dinucleotide | (AC)^9 |
| contig09072 | EZ125291 | Dinucleotide | (AT)^9 |
| contig15833 | EZ132052 | Dinucleotide | (GT)^13 |
| contig10308 | EZ126527 | Dinucleotide | (AC)^7 |
| contig04327 | EZ120546 | Dinucleotide | (AT)^8 |
| contig15378 | EZ131597 | Dinucleotide | (AC)^9 |
| contig16457 | EZ132676 | Dinucleotide | (AT)^8 |
| contig05063 | EZ121282 | Dinucleotide | (AC)^9 |
| contig05175 | EZ121394 | Dinucleotide | (AT)^7 |
| contig15353 | EZ131572 | Dinucleotide | (AT)^7 |
| contig24199 | EZ140418 | Dinucleotide | (AT)^7 |
| contig24337 | EZ140556 | Dinucleotide | (AC)^7 |
| contig06767 | EZ122986 | Dinucleotide | (AG)^13 |
| contig03525 | EZ119744 | Dinucleotide | (AT)^7 |
| contig16272 | EZ132491 | Dinucleotide | (GT)^7 |
| contig13648 | EZ129867 | Dinucleotide | (AT)^7 |
| contig16894 | EZ133113 | Dinucleotide | (AT)^8 |
| contig17580 | EZ133799 | Dinucleotide | (AC)^7 |
| contig23273 | EZ139492 | Dinucleotide | (AC)^8 |
| contig19982 | EZ136201 | Dinucleotide | (AT)^7 |
| contig21016 | EZ137235 | Dinucleotide | (AC)^7 |
| contig08559 | EZ124778 | Dinucleotide | (AC)^9 |
| contig17739 | EZ133958 | Dinucleotide | (AT)^8 |
| contig21821 | EZ138040 | Dinucleotide | (AT)^7 |
| contig24002 | EZ140221 | Dinucleotide | (AC)^7 |
| contig22505 | EZ138724 | Dinucleotide | (AC)^7 |
| contig07803 | EZ124022 | Dinucleotide | (AC)^7 |
| contig13591 | EZ129810 | Dinucleotide | (AC)^7 |
| contig13110 | EZ129329 | Dinucleotide | (GT)^7 |
| contig14355 | EZ130574 | Dinucleotide | (AT)^8 |
| contig22911 | EZ139130 | Dinucleotide | (AT)^7 |
| contig24353 | EZ140572 | Dinucleotide | (AT)^7 |
| contig02475 | EZ118694 | Dinucleotide | (AC)^7 |
| contig08361 | EZ124580 | Dinucleotide | (AC)^7 |
| contig24318 | EZ140537 | Dinucleotide | (AC)^7 |
| contig00238 | EZ116457 | Dinucleotide | (AC)^7 |
| contig01728 | EZ117947 | Dinucleotide | (AC)^10 |
| contig11107 | EZ127326 | Dinucleotide | (GT)^7 |
| contig05530 | EZ121749 | Dinucleotide | (AG)^8 |
| contig21718 | EZ137937 | Dinucleotide | (AT)^7 |
| contig02929 | EZ119148 | Dinucleotide | (CT)^8 |
| contig10178 | EZ126397 | Dinucleotide | (GT)^7 |
| contig09671 | EZ125890 | Dinucleotide | (CT)^9 |
| contig22789 | EZ139008 | Dinucleotide | (AT)^9 |
| contig11050 | EZ127269 | Dinucleotide | (AC)^8 |
| contig20185 | EZ136404 | Dinucleotide | (AC)^7 |
| contig23858 | EZ140077 | Dinucleotide | (GT)^8 |
| contig04390 | EZ120609 | Dinucleotide | (AC)^8 |
| contig05212 | EZ121431 | Dinucleotide | (AC)^8 |
| contig24368 | EZ140587 | Dinucleotide | (GT)^7 |
| contig20183 | EZ136402 | Dinucleotide | (AC)^12 |
| contig20458 | EZ136677 | Dinucleotide | (AC)^8 |
| contig21941 | EZ138160 | Dinucleotide | (AT)^8 |
| contig22366 | EZ138585 | Dinucleotide | (AC)^7 |
| contig10998 | EZ127217 | Dinucleotide | (AC)^9 |
| contig23178 | EZ139397 | Dinucleotide | (AT)^7 |
| contig21948 | EZ138167 | Dinucleotide | (AT)^7 |
| contig10178 | EZ126397 | Dinucleotide | (GT)^8 |
| contig01170 | EZ117389 | Dinucleotide | (AT)^9 |
| contig07371 | EZ123590 | Dinucleotide | (AC)^8 |
| contig24236 | EZ140455 | Dinucleotide | (AT)^8 |
| contig04897 | EZ121116 | Trinucleotide | (ATT)^7 |
| contig15735 | EZ131954 | Trinucleotide | (AAT)^8 |
| contig15035 | EZ131254 | Trinucleotide | (GAT)^8 |
| contig02759 | EZ118978 | Trinucleotide | (GAT)^7 |
| contig09196 | EZ125415 | Trinucleotide | (AAC)^9 |
| contig05363 | EZ121582 | Trinucleotide | (ACT)^7 |
| contig17813 | EZ134032 | Trinucleotide | (AAC)^7 |
| contig20382 | EZ136601 | Trinucleotide | (ATT)^7 |
| contig14182 | EZ130401 | Trinucleotide | (AAC)^7 |
| contig16875 | EZ133094 | Trinucleotide | (AAT)^7 |
| contig10168 | EZ126387 | Trinucleotide | (AAT)^7 |
| contig02728 | EZ118947 | Trinucleotide | (AAC)^8 |
| contig06344 | EZ122563 | Trinucleotide | (ATT)^8 |
| contig21285 | EZ137504 | Trinucleotide | (AAC)^7 |
| contig21064 | EZ137283 | Trinucleotide | (GTT)^7 |
| contig04736 | EZ120955 | Trinucleotide | (GAT)^11 |
| contig16191 | EZ132410 | Trinucleotide | (AAT)^7 |
| contig00128 | EZ116347 | Trinucleotide | (AAT)^7 |
| contig03158 | EZ119377 | Trinucleotide | (GTT)^7 |
| contig23947 | EZ140166 | Trinucleotide | (AAT)^7 |
| contig24247 | EZ140466 | Trinucleotide | (AAC)^7 |
| contig24230 | EZ140449 | Trinucleotide | (GCT)^8 |
| contig18570 | EZ134789 | Trinucleotide | (AGG)^7 |
| contig11269 | EZ127488 | Trinucleotide | (ATT)^7 |
| contig07629 | EZ123848 | Trinucleotide | (AAT)^7 |
| contig09196 | EZ125415 | Trinucleotide | (GGT)^7 |
| contig11320 | EZ127539 | Trinucleotide | (ATT)^7 |
| contig14907 | EZ131126 | Trinucleotide | (AAC)^8 |
| contig14737 | EZ130956 | Trinucleotide | (AAT)^7 |
| contig21041 | EZ137260 | Trinucleotide | (AAC)^8 |
| contig10073 | EZ126292 | Trinucleotide | (AAC)^7 |
| contig23653 | EZ139872 | Trinucleotide | (GTT)^9 |
| contig22689 | EZ138908 | Trinucleotide | (AAC)^8 |
| contig01714 | EZ117933 | Trinucleotide | (AAG)^10 |
| contig21029 | EZ137248 | Trinucleotide | (GTT)^7 |
| contig14791 | EZ131010 | Tetranucleotide | (ACAT)^7 |
| contig21972 | EZ138191 | Tetranucleotide | (ACAT)^7 |
| contig14523 | EZ130742 | Tetranucleotide | (ATGT)^7 |
| contig08778 | EZ124997 | Tetranucleotide | (ATGT)^9 |
| contig17444 | EZ133663 | Tetranucleotide | (ACAT)^7 |
| contig22789 | EZ139008 | Tetranucleotide | (ATGT)^8 |
| contig12964 | EZ129183 | Tetranucleotide | (ACAT)^7 |
| contig08369 | EZ124588 | Hexanucleotide | (GTCTTT)^22 |
| contig10732 | EZ126951 | Hexanucleotide | (AACATT)^7 |
| contig10732 | EZ126951 | Hexanucleotide | (AACATT)^7 |
