# Supplementary material for: Sympatric ecological speciation meets pyrosequencing: sampling the transcriptome of the apple maggot Rhagoletis pomonella
Source: BMC Genomics. 2009 Dec 27;10:633. doi: 10.1186/1471-2164-10-633 (PMC2807884; doi:10.1186/1471-2164-10-633)
Supplement: Additional file 7 — Table of transcript gain in the lineage leading to the Schizophora since the last common ancestor of mosquitoes, Rhagoletis, and Drosophila. D. melanogaster annotation denotes the Celera Genome number of the locus with the closest match and locus name where known. Contig is the R. pomonella contig number followed by the TSA accession number. [file 1471-2164-10-633-S7.DOCX]

**Additional file 7**

| ***D. melanogaster* annotation** | | **Note** | ***Rhagoletis* Contig.** | **TSA Acc.** |
| --- | --- | --- | --- | --- |
| Gene product (unknown function) | CG15201 | novel to Schizophora | contig24366 | EZ140585 |
| Gene product (unknown function) | CG13144 | novel to Schizophora | contig01397 | EZ117616 |
| Gene product (unknown function) | CG1552 | novel to Schizophora | contig10054 | EZ126273 |
| Gene product (unknown function) | CG12057 | novel to Schizophora | contig09054 | EZ125273 |
| Tetraspannin 42Eh | CG12844 |  | contig09325 | EZ125544 |
| Gene product (unknown function) | CG15406 |  | contig04014 | EZ120233 |
| LP09690p | CG6839 |  | contig01753 | EZ117972 |
| Serendipity delta | CG17958 |  | contig04040 | EZ120259 |
